# A Novel Mutation of *OsPPDKB*, Encoding Pyruvate Orthophosphate Dikinase, Affects Metabolism and Structure of Starch in the Rice Endosperm

**DOI:** 10.3390/ijms19082268

**Published:** 2018-08-02

**Authors:** Long Zhang, Linglong Zhao, Lingshang Lin, Lingxiao Zhao, Qiaoquan Liu, Cunxu Wei

**Affiliations:** 1 Key Laboratory of Crop Genetics and Physiology of Jiangsu Province, Key Laboratory of Plant Functional Genomics of the Ministry of Education, Yangzhou University, Yangzhou 225009, China; zhanglong@yzu.edu.cn (L.Z.); 18362821828@163.com (L.Z.); 18252713442@163.com (L.L.); d150106@yzu.edu.cn (L.Z.); qqliu@yzu.edu.cn (Q.L.); 2 Co-Innovation Center for Modern Production Technology of Grain Crops of Jiangsu Province, Joint International Research Laboratory of Agriculture & Agri-Product Safety of the Ministry of Education, Yangzhou University, Yangzhou 225009, China

**Keywords:** rice, pyruvate orthophosphate dikinase, starch, floury endosperm, amyloplast development

## Abstract

Starch, as a main energy storage substance, plays an important role in plant growth and human life. Despite the fact that several enzymes and regulators involved in starch biosynthesis have been identified, the regulating mechanism of starch synthesis is still unclear. In this study, we isolated a rice floury endosperm mutant *M14* from a mutant pool induced by ^60^Co. Both total starch content and amylose content in *M14* seeds significantly decreased, and starch thermal and pasting properties changed. Compound starch granules were defected in the floury endosperm of *M14* seeds. Map-based cloning and a complementation test showed that the floury endosperm phenotype was determined by a gene of *OsPPDKB*, which encodes pyruvate orthophosphate dikinase (PPDK, EC 2.7.9.1). Subcellular localization analysis demonstrated that PPDK was localized in chloroplast and cytoplasm, the *chOsPPDKB* highly expressed in leaf and leaf sheath, and the *cyOsPPDKB* constitutively expressed with a high expression in developing endosperm. Moreover, the expression of starch synthesis-related genes was also obviously altered in *M14* developing endosperm. The above results indicated that PPDK played an important role in starch metabolism and structure in rice endosperm.

## 1. Introduction

In cereal endosperm, starch is synthesized in amyloplasts and forms starch granules. Mature seeds of cereals such as rice, maize and wheat contain large amounts of starch. It provides a substrate for seed germination and seedling growth. In addition, endosperm starch makes an important contribution to the human diet throughout the world [1].

Starch consists of two major components, amylose and amylopectin. Amylose is a linear polymer made of α-1,4-linked d-glucopyranosyl units, whereas amylopectin is a highly branched macromolecule [2]. The enzymology of starch synthesis has been well studied. ADP-glucose (ADPG) is produced by ADP-glucose pyrophosphorylase (AGPase) and is then used as a substrate to initiate amylose and amylopectin synthesis and elongation. Granule-bound starch synthase I (GBSSI) is an amylose-specific starch synthase, whereas starch synthases (SSI, SSII, SSIII, and SSIV), starch branching enzymes (BEI and BEII), and starch debranching enzymes (isoamylase (ISA) and pullulanase (Pul)) are responsible for amylopectin synthesis [1]. In rice, the mutations in the above starch synthesis key enzymes cause abnormal characteristics of endosperm starch with opaque-seed phenotype including shrunken, floury and white-core endosperm. For example, mutations in AGPase small subunit OsAGPS2 and large subunit OsAGPL2 decrease the synthesis of ADPG and form small and round starch granules [3]. Mutation in *ISA1* produces a sugary endosperm and accumulates abundant phytoglycogen instead of normal starch granules [4]. The SSIIIa-deficient mutant causes a floury endosperm and reduces the content of long branch-chains of amylopectin [5,6]. The *amylose-extender* (*ae*) mutant lacking BEIIb produces a white-core endosperm and alters the fine structure of amylopectin [7].

In addition, some mutations in genes that are not directly involved in starch metabolism also affect starch synthesis. *FLO2* (*FLOURY ENDOSPERM2*), encoding a tetratricopeptide repeat motif-containing protein, affects the starch quality by regulating the expression of starch synthesis-related genes [8,9]. *FLO6* contains a CBM48 domain in the C-terminal and regulates the starch synthesis and starch granule development by interacting with ISA1 [10]. A domain of unknown function 1338 (DUF1338) superfamily member *FLO7* influences the amylose content and amylopectin structure in the outer endosperm [11]. *FLO8*, encoding the UDP-glucose pyrophosphorylase 1 (Ugp1), modulates the starch synthesis and structure [12]. Besides the *FLOURY* genes, other genes can also cause floury endosperm phenotype after loss of function, such as *Grain Incomplete Filling1* (*GIF1*) encoding a cell wall invertase, *Fructose-6-phosphate 1-phosphotransferase* (*PFP*) encoding a fructose-6-phosphate 1-phosphotransferase β subunit, and *Glutelin Precursor Accumulation3* (*GPA3*) encoding a plant-specific kelch-repeat protein [13,14,15]. Therefore, the genes involved in carbon partitioning, carbon flux and protein body development may also affect the development of rice kernel and starch metabolism.

Pyruvate orthophosphate dikinase (PPDK, EC 2.7.9.1) catalyzes the interconversion of pyruvate, ATP, and Pi with phosphoenolpyruvate, AMP, and PPi [16]. PPDK in C_4_ plants is most well known as a photosynthetic enzyme, which catalyzes the formation of CO_2_ acceptor phosphoenolpyruvate from pyruvate [17]. The activity of chloroplastic PPDK can be controlled by reversible phosphorylation of a threonine residue [18]. Maize, a typical C_4_ species, contains three PPDK genes, *C_4_ppdkZm1*, *cyppdkZm1* and *cyppdkZm2* [19]. During the accumulation of zein protein in developing endosperm, *cyPPDKZm1* can be regulated by the *O*_2_ (*Opaque2*) gene [20]. In most C_3_ tissues, PPDK is either not present or at very low abundance [21]. In rice, there are only two genes encoding PPDK, one encoding OsPPDKA and another producing two transcripts of C_4_-type chloroplastic PPDK (chOsPPDKB) and cytosolic PPDK (cyOsPPDKB) [22,23]. A corresponding histochemical analysis of β-glucuronidase (*GUS*) gene driven by cytosolic OsPPDKB promoter is conducted in rice. The GUS activity shows a high level in developing kernels, but a low level in the flowers and roots [24]. The T-DNA insertional mutant of *OsPPDKB* shows a white-core endosperm, indicating the essential role of *OsPPDKB* in modulating the carbon flow for starch and lipid synthesis [16]. Recently, a new *cyOsPPDKB* mutant Suweon 542 with a milky-white floury endosperm has been identified and characterized [25]. However, the effect of *OsPPDKB* mutation on starch development, starch physicochemical properties and starch metabolism are still unknown.

In the present study, we characterized a floury endosperm mutant, *M14*. Map-based cloning and complementation test revealed that the mutant phenotype was caused by *OsPPDKB*, which is a new allele of *FLO4* [16] from rice variety Kitaake. Meanwhile, the starch development, starch physicochemical properties and expression of starch synthesis-related genes in *M14* and its wild type (WT) were investigated and compared. Our data showed a vital importance of OsPPDKB in starch metabolism and structure in rice endosperm development.

## 2. Results

### 2.1. Phenotypic Analysis of M14

**Figure 1:**
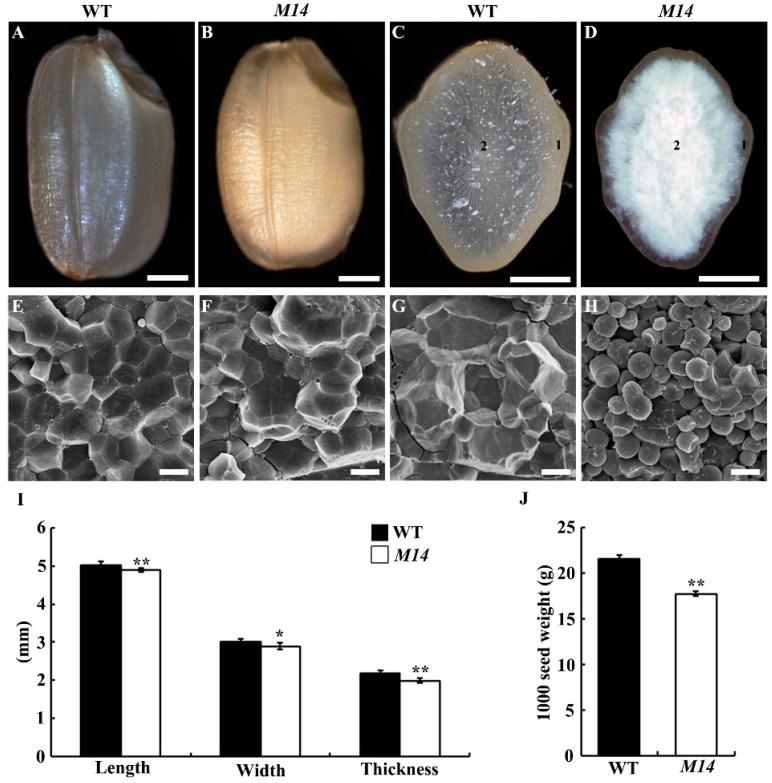
Phenotype analyses of *M14* seeds. (**A**,**B**) The appearance of brown rice of WT and *M14*. (**C**,**D**) Transverse sections of WT and *M14* seed. Bars = 1 mm (**A**–**D**). (**E**–**H**) Scanning electron microscope observation of WT and *M14* seeds. (**E**,**F**) represent the region of “1” and “2” in WT endosperm, respectively, and (**G**,**H**) represent the region of “1” and “2” in *M14* endosperm, respectively. Bars = 5 μm (**E**–**H**). (**I**) Seed length, width, and thickness of WT and *M14* (*n* = 20). (**J**) 1000-seed weight of WT and *M14*. Data are given as means ± SD (*n* = 3). ** and * indicate significant differences between WT and *M14* at *p* < 0.01 and *p* < 0.05 by Student’s *t* test.

The *M14* with a floury endosperm phenotype was identified from a ^60^Co-irradiated mutant pool of *japonica* rice variety Kitaake, and displayed no visible differences from WT plants at the vegetative stage. The brown rice of *M14* exhibited an opaque endosperm when compared with WT seeds (Figure 1A,B). The seed cross section displayed that the inner endosperm of *M14* appeared floury, while the peripheral endosperm was as transparent as the WT (Figure 1C,D). Scanning electron microscope (SEM) analysis showed that the floury endosperm of *M14* comprised small, round and loosely packed compound starch granules, which was different from the transparent endosperm of *M14* and WT with densely packed, polyhedral compound starch granules (Figure 1E–H). Compared with WT, the seed length, width and thickness of *M14* significantly decreased (Figure 1I). Therefore, the 1000-seed weight of *M14* was markedly reduced by 11.4% (Figure 1J).

### 2.2. The Abnormal Morphology of Compound Starch Granules in M14 Endosperm

**Figure 2:**
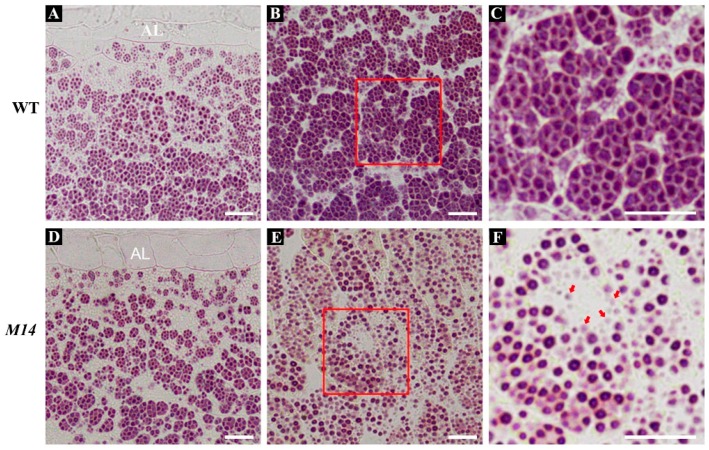
The formation of abnormal compound starch granules in *M14* endosperm. (**A**–**F**) Semi-thin sections of WT (**A**–**C**) and *M14* (**D**–**F**) kernels at 9 DAF are stained with I_2_-KI. (**A**,**D**) and (**B**,**E**) represent the outer and inner of endosperm, respectively. (**C**,**F**) represent the magnified region indicated by red outline in WT and *M14* endosperm, respectively. Red arrowheads in (**F**) indicate abnormal weakly stained starch granules. AL, aleurone layer. Bars = 20 μm.

To observe the morphology of compound starch granules, the developing endosperm of WT and *M14* at 9 days after fertilization (DAF) was prepared into semi-thin sections. I_2_-KI staining displayed that amyloplasts in the outer endosperm of *M14* and WT developed well and filled with densely packed, polyhedral starch granules (Figure 2A,D). The inner endosperm of WT showed normal compound starch granules (Figure 2B,C), however, that *of M14* contained a large number of single and abnormal weakly stained starch granules (Figure 2E,F), indicating that *M14* mutation changed the formation of normal compound starch granules in rice endosperm.

### 2.3. Physicochemical Properties of Starch from M14 Seeds

**Figure 3:**
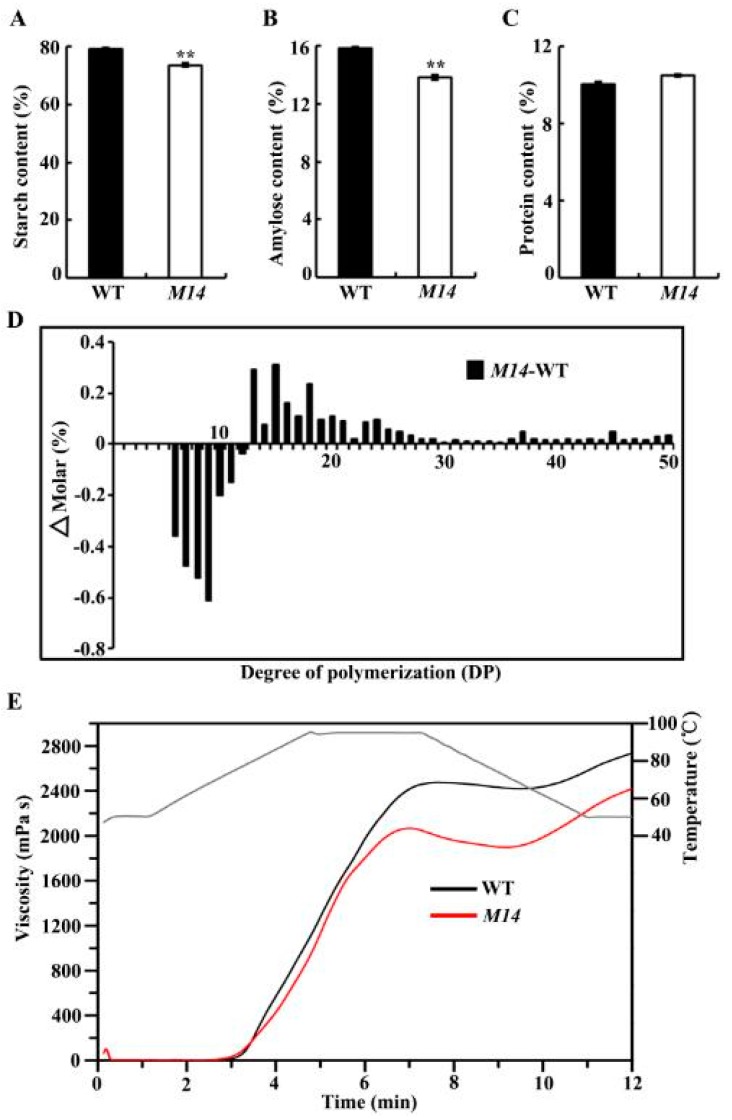
Components of seed and starch and pasting properties of starch. (**A**–**C**) Starch, amylose and protein contents in WT and *M14*. All data are given as means ± SD (*n* = 3). ** indicate significant differences between WT and *M14* at *p* < 0.01 by Student’s *t* test. (**D**) Differences in chain length distributions of amylopectin between WT and *M14*. (**E**) Pasting properties of starch from WT (black line) and *M14* (red line) endosperm. The gray line indicates the temperature changes during the measurements.

The seeds of WT had a starch content of 79.1%, whereas the *M14* had a significantly decreased starch content (73.6%) (Figure 3A). The amylose content decreased in *M14* (13.8%) compared with WT (15.8%) (Figure 3B), but protein content slightly increased (10.5% vs. 10.0%) (Figure 3C). The amylopectin fine structure analysis showed that the short branch-chains with degree of polymerization (DP) 6–12 increased in *M14*, whereas the intermediate branch-chains with DP 13–50 decreased compared with WT (Figure 3D). The thermal properties of WT and *M14* starches were measured by differential scanning calorimetry (DSC), and their thermal parameters are shown in Table 1. The gelatinization onset temperature, peak temperature, and conclusion temperature of *M14* starch were all about 2 °C lower than those of WT starch. The pasting properties of WT and *M14* starches determined by rapid visco analyzer (RVA) are presented in Figure 3E, and their pasting parameters are given in Supplemental Table S1. The *M14* starch had significantly lower peak viscosity, hot viscosity, and final viscosity than WT starch. In conclusion, the physicochemical characteristics of *M14* starch changed significantly compared with WT starch.

### 2.4. Map-Based Cloning of the Gene Responsible for the M14 Mutation

**Figure 4:**
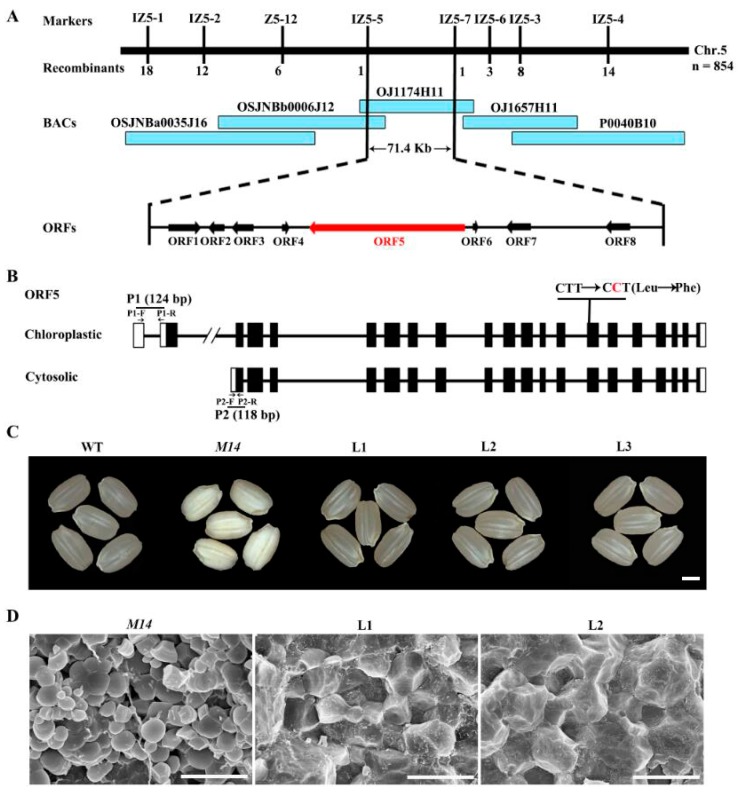
Map-based cloning and complementation of *M14*. (**A**) Fine mapping of the *OsPPDKB* locus (red arrow). The *OsPPDKB* locus is mapped to a 71.4 kb region on chromosome 5, which contains eight predicted ORFs. (**B**) Structure of the *OsPPDKB* gene and positions of mutant site and qRT-PCR primers. The sequence of P1 (124 bp) was amplificated by specific primers P1-F and P1-R and the sequence of P2 (118 bp) was amplificated by specific primers P2-F and P2-R. (**C**,**D**) Complementation test of the *OsPPDKB* gene completely rescues the seed appearance (**C**) and compound starch granule arrangement (**D**) of *M14*. Bars = 2 mm (**C**) and 10 μm (**D**).

For genetic analysis, an F_2_ population was produced from reciprocal crosses between WT and *M14*. All F_1_ seeds displayed WT phenotype, and the F_2_ population exhibited a 3:1 segregation ratio (497 normal: 156 floury; χ^2^ = 0.43 < χ^2^_0.05,1_ = 3.84). Therefore, the floury phenotype in the *M14* was controlled by a recessive nuclear gene. In order to identify the mutation locus controlling the *M14* phenotype, the approach of map-based cloning was adopted. An F_2_ mapping population with approximately 5000 progeny was created by crossing the *M14* with the wide-compatibility *indica* variety Dular. For bulk-segregant analysis (BSA), two DNA pools were generated from 10 F_2_ floury endosperm and 10 normal endosperm individuals, respectively. These two pools were genotyped with 170 selected insertion-deletion (InDel) markers for rough mapping. The *M14* mutation locus was initially mapped between InDel marker IZ5-1 and IZ5-4 on the long arm of chromosome 5. On this basis, 854 recessive individuals were selected and detected by the two pairs of primers to screen recombinants. To further narrow down the locus, six new InDel markers were developed. The number of recombinants gradually decreased as primers were closer to the target gene (Figure 4A). The *M14* mutation locus was finally mapped to a 71.4 kb region between InDel markers IZ5-5 and IZ5-7. The 71.4 kb region on the BAC clone OJ1174H11 contained 8 putative ORFs (http://rapdb.dna.affrc.go.jp) (Figure 4A). The genome DNA of 8 ORFs was all sequenced and only one nucleotide substitution of thymidine (T) to cytosine (C) was found in the *Os05g0405000*, which encodes pyruvate orthophosphate dikinase (PPDK) (Figure 4B). Amino acid sequence analysis indicated that the single nucleotide mutation caused Leu-637 replacement by Phe (Figure 4B). The expression level of the *cyOsPPDKB* in *M14* was comparable to the WT at 9 DAF endosperm (Supplemental Figure S1).

In rice, the *OsPPDKB* gene contains two transcripts, *chOsPPDKB* and *cyOsPPDKB*, and the *cyOsPPDKB* expressed at a high level in developing kernels [24]. To confirm that *Os05g0405000* was responsible for the *M14* mutation, the pUbi:cyOsPPDKB was introduced into the *M14* to complement the floury phenotype. Positive transgenic lines expressing pUbi:cyOsPPDKB were identified by PCR analysis (Supplemental Figure S2). The seed phenotype and compound starch granule morphology in three positive transgenic lines were rescued (Figure 4C,D). Therefore, *Os05g0405000* was the gene responsible for the *M14* phenotype.

### 2.5. Expression Pattern and Subcellular Localization of OsPPDKB

**Figure 5:**
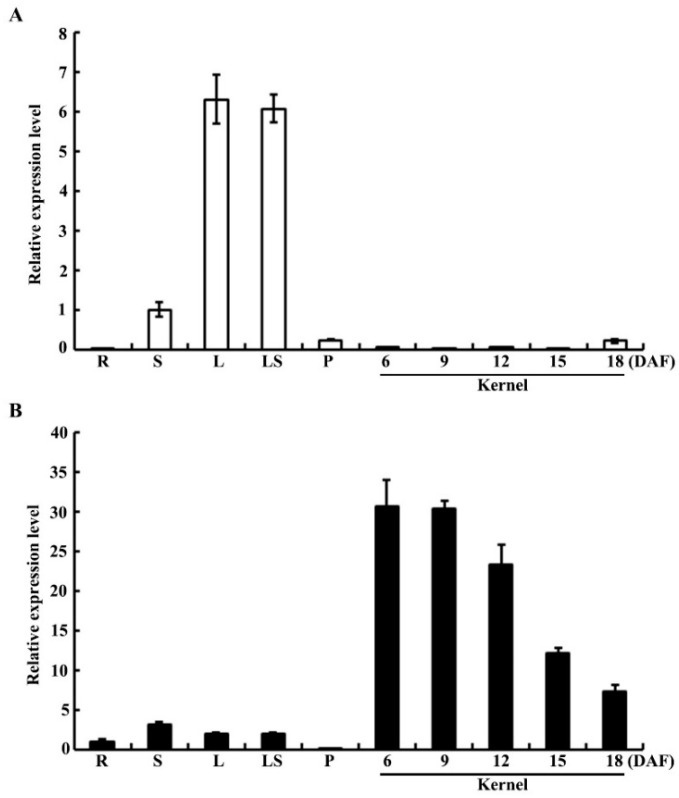
Expression pattern of *OsPPDKB*. (**A**) Expression analysis of the *chOsPPDKB* in various tissues and developing kernels of WT. (**B**) Expression analysis of the *cyOsPPDKB* in various tissues and developing kernels of WT. R, root; S, stem; L, leaf; LS, leaf sheath; P, panicle. *Actin1* is used as an internal control. All data are given as means ± SD (*n* = 3).

We examined the *chOsPPDKB* and *cyOsPPDKB* expression patterns with two pairs of specific primers (P1-F/P1-R and P2-F/P2-R in Figure 4B) by quantitative real-time PCR (qRT-PCR). In WT plants, the *chOsPPDKB* expressed in the stem, leaf, leaf sheath and panicle, with the highest expression level in leaf and leaf sheath (Figure 5A). In contrast, the expression of *cyOsPPDKB* was detected in the root, stem, leaf, leaf sheath and developing kernels, with the highest expression in developing kernels (Figure 5B). During kernel development, the expression of *cyOsPPDKB* was high at the early stage and then gradually declined (Figure 5B). The expression trend of *cyOsPPDKB* in endosperm was similar to those of *OsAGPL3*, *OsAGPS2b*, *OsGBSSI*, *OsSSIIa*, *OsSSIIIa*, *OsBEI*, *OsBEIIb*, and *OsISA2*, demonstrating the coexpression of *cyOsPPDKB* and these genes [26].

**Figure 6:**
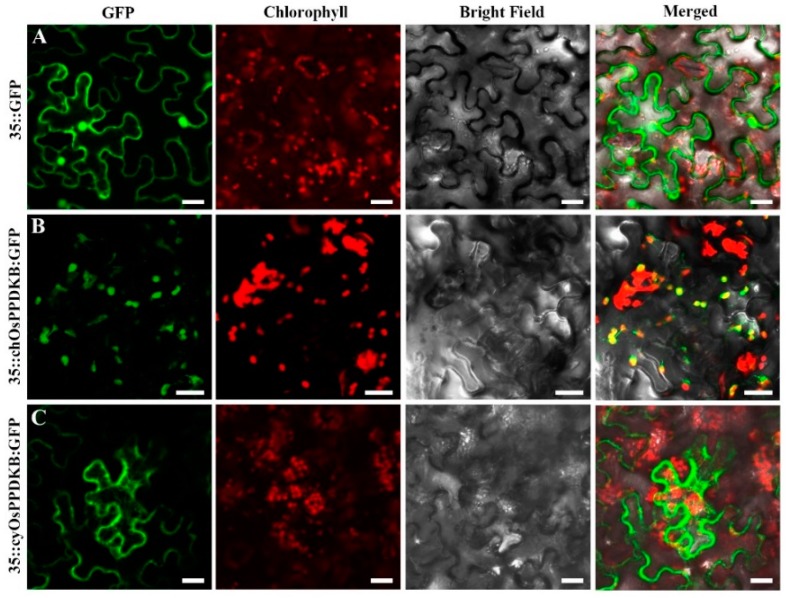
Subcellular localization of OsPPDKB-GFP fusion proteins in *Nicotiana benthamiana* leaf. (**A**) The free GFP serves as a control. (**B**) The chOsPPDKB-GFP fusion protein is localized in the chloroplasts. (**C**) The cyOsPPDKB-GFP fusion protein is localized in the cytoplasm. Bars = 20 μm.

To identify the subcellular localization of OsPPDKB, two GFP fusions, chOsPPDKB-GFP and cyOsPPDKB-GFP, were transiently expressed in *Nicotiana benthamiana* leaves. The free GFP protein was widely distributed in the nuclei and cytoplasm (Figure 6A), while the chOsPPDKB-GFP proteins co-localized exclusively with chlorophyll autofluorescence signals (Figure 6B) and the cyOsPPDKB-GFP was localized in cytoplasm, which displayed a dispersed distribution throughout the leaves (Figure 6C).

## 3. Discussion

Starch, as the main storage material, occupies most of the volume of rice endosperm [27]. Defects in rice starch synthesis affect kernel development and weight, and further change the physicochemical properties of starch. In this study, we isolated and identified a rice floury endosperm mutant, *M14*. Map-based cloning and complementation test revealed that the mutant phenotype was controlled by the gene of *OsPPDKB*. The function knockout mutations of *FLO4*/*OsPPDKB* display a white-core endosperm phenotype [16]. Despite both *flo4* and *M14* displaying a similar opaque appearance in seeds, the *M14* showed a more serious defective phenotype with obviously decreased seed weight, starch content and amylose content (Figure 1D,J and Figure 3A,B) than *flo4* [16]. Similar results are reported in the *flo2* mutant. The seeds in *flo2* mutant *CNY8-1* exhibit an opaque appearance with whole floury endosperm, while the *flo2* mutant *flo*(*a*) contains only a white-core endosperm in the center of seeds [9,28]. This phenotype difference may be due to genetic background and environmental effects [9].

**Figure 7:**
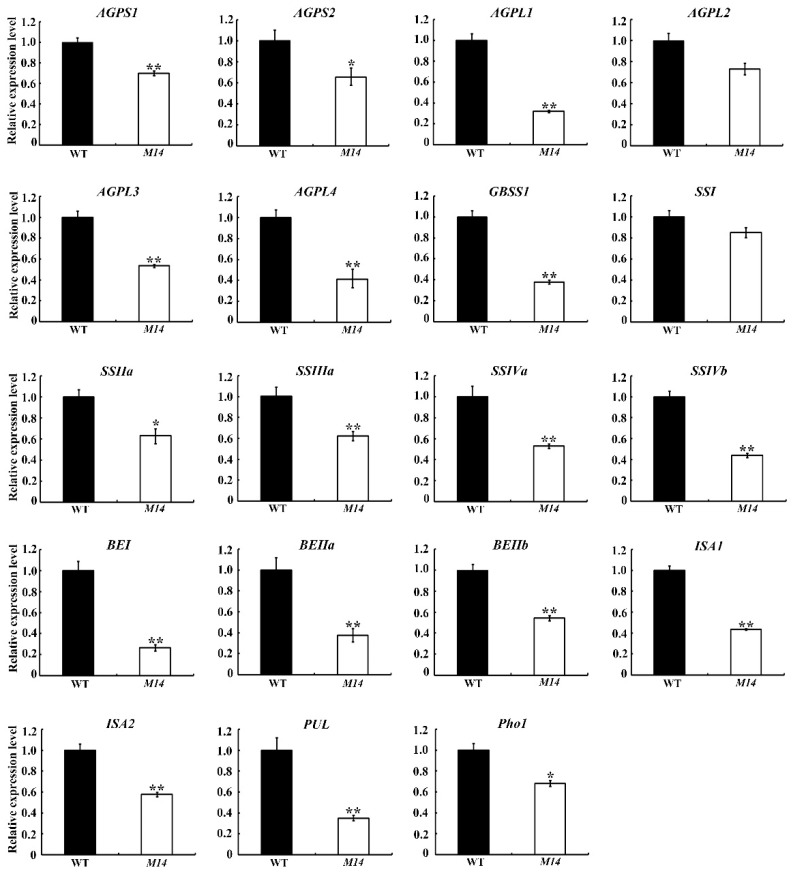
Expression of starch biosynthesis-related genes in WT and *M14*. Total RNA extracted from 9 DAF endosperm is used for qRT-PCR analysis. ADP glucose pyrophosphorylase small subunit (AGPS1 and AGPS2) and large subunit (AGPL1, AGPL2, AGPL3, and AGPL4); granule-bound starch synthase I (GBSSI); soluble starch synthase I, IIa, IIIa, IVa, and IVb (SSI, SSIIa, SSIIIa, SSIVa, and SSIVb); branching enzyme I, IIa, and IIb (BEI, BEIIa, and BEIIb); isoamylase isozymes (ISA1 and ISA2); pullulanase (PUL); plastidial phosphorylase (Pho1). The expression level of every target gene in WT is set at 1.0. All data are given as means ± SD (*n* = 3). ** and * indicate significant differences between WT and *M14* at *p* < 0.01 and *p* < 0.05 by Student’s *t* test.

**Figure 8:**
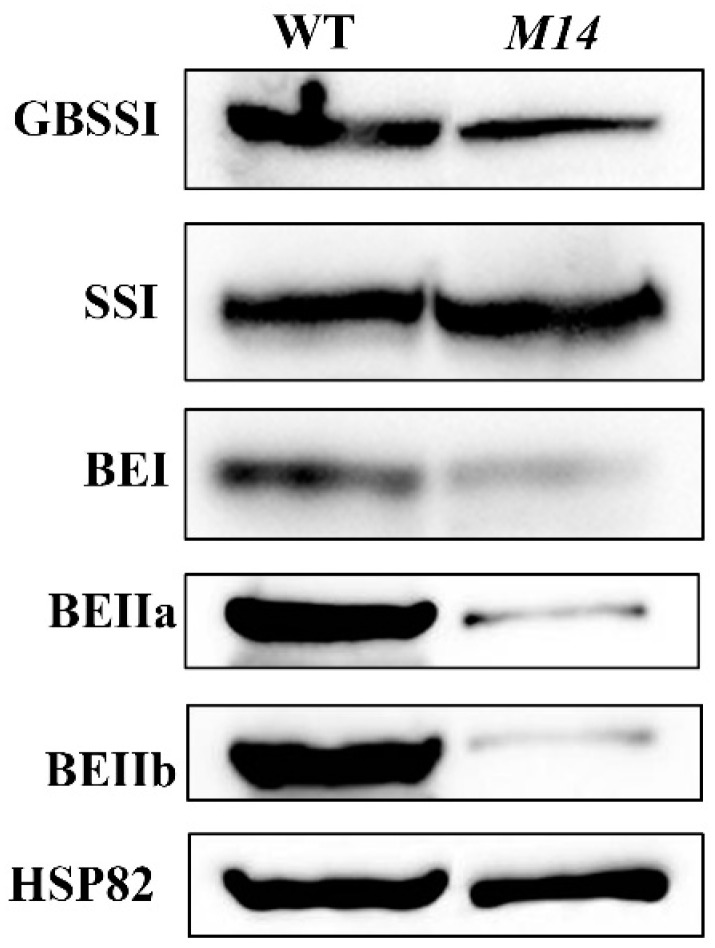
Accumulation of proteins involved in starch synthesis in WT and *M14* endosperm at 9 DAF. Anti-GBSSI, anti-SSI, anti-BEI, anti-BEIIa and anti-BEIIb are used for the immunoblot analysis. Anti-HSP82 is used for the loading control.

Amylose is an important factor affecting the physicochemical properties of starch. The mutation of *OsPPDKB* led to a significant down-regulation of GBSSI expression (Figure 7 and Figure 8), and resulted in the reduction of amylose synthesis, with amylose content of *M14* estimated at 13.8%, approximately a 12.6% reduction compared to WT (Figure 3B). Compared with WT starch, *M14* starch exhibited a lower gelatinization peak temperature (Table 1). The gelatinization peak temperature correlates positively with amylose content [29], which is consistent with the present study. The low amylose content of *M14* led to different RVA profiles, including the PV, HV, and FV (Figure 3E; Supplemental Table S1). *GBSSI*, *SSIIa* and *PUL* together with *BEI* and *BEIIb* affect the starch pasting properties in rice [30,31,32]. Indeed, the expression of these starch synthesis genes significantly reduced in *M14* (Figure 7) and, as a result, the pasting properties of *M14* starch also changed. Floury endosperm with low seed hardness, low starch damage, and fine particle size is suitable for dry milling, such as the *cyOsPPDKB* mutant Suweon 542 [25]. In this study, another novel *OsPPDKB* mutant *M14* with floury endosperm was identified, and the physicochemical properties of starch were further analyzed, which would provide a fine flour with a special property for quality breeding.

This seed phenotype of *M14* was similar to that of rice *ssg1/ssg2/ssg3/beiib*, *flo6*, *ssg4* and *ss3ass4b* mutants having a floury endosperm. The endosperm of the *ssg4* mutant has abundant enlarged amyloplasts containing many starch subgranules, and the mutations in *SSG1/BEIIb* and *FLO6* produce small and abnormal starch granules [10,33]. Interestingly, a large number of smaller, single starch granules were observed in the inner of *M14* endosperm (Figure 2E,F), and were similar to those of the *ss3ass4b* mutant [34]. In addition, the expression of *SSIIIa* and *SSIVb* were significantly reduced in *M14* endosperm (Figure 7). The difference in starch morphology among *M14*, *ssg1/beiib*, *flo6* and *ssg4* might be due to the different effects of mutation on amylose content and amylopectin structure. In maize, the granule diameter distribution in the PPDK-deficient lines is shifted toward smaller particles [35], which is similar to the results observed in the *M14* endosperm. Taken together, OsPPDKB played an important role in the formation of compound starch granules.

A floury seed phenotype is also conditioned by PPDK deficiency in maize [35], although it has clear distinctions from rice. Specifically, a single mutant lacking function of rice *OsPPDKB* caused the phenotype, whereas maize *pdk1/C*_4_*ppdkZm1* is a minor contributor to total endosperm PPDK, and the floury phenotype is observed only when both the *pdk1/C_4_ppdkZm1* and *pdk2/cyppdkZm1* functions are eliminated. The mutation of *chOsPPDKB* in *M14* did not significantly affect the growth of the seedlings (Supplemental Figure S3), while loss-of-function mutant of *chOsPPDKB* homolog (*pdk1*/*C_4_ppdkZm1*) in maize is seedling lethal. One hypothesis is that other genes with a similar function in photosynthesis might offset the loss caused by mutation in the *chOsPPDKB* gene in *M14*. Unlike rice, there are no significant differences in amylose content and the fine structure of amylopectin in maize PPDK-deficient transgenic seeds [35], which may be due to more storage protein rather than starch in maize endosperm. Owing to that the expression of most genes in the starch synthesis pathway reduced in *M14*, the amylose content and amylopectin structure changed significantly (Figure 3B,D and Figure 7).

Three hypotheses for PPDK functions in endosperm were reported, (i) participation in the process of gluconeogenesis and provision of hexose for starch synthesis, (ii) providing pyruvate for the synthesis of lipids, and (iii) regulating the sucrose synthase by controlling the homoeostasis of PPi [16,35,36]. The *M14* had a single nucleotide T to C substitution in the coding region of *cyOsPPDKB*, leading to the Leu-637 being replaced by Phe. The single amino acid substitution did not affect the expression of *cyOsPPDKB* in *M14* endosperm, which was different from the Suweon 542 mutant [25]. This residue was just located in the PEP-utilizers domain (Supplemental Figure S4A). Amino acid sequence analysis revealed that the Leu-637 residue was highly conserved in the PPDK among different plant species (Supplemental Figure S4B). PPDK catalyzes the interconversion of ATP, Pi, and pyruvate to AMP, PPi, and PEP. The pyruvate kinase (PK) catalyzes PEP to pyruvate, which is an irreversible reaction. Therefore, the Leu-637 mutation of *OsPPDKB* in *M14* might affect the reaction, that pyruvate and Pi in the cytosol of developing endosperm is converted into PEP and PPi. The reaction of PEP to pyruvate is normal. In this case, the recycling of PEP into hexose pools may be reduced, while the concentration of PPi in the cytosol may be increased. The PEP participates in the gluconeogenesis and provides hexose for starch biosynthesis [37]. The PPi is utilized as an alternative energy donor for the conversion of imported sucrose into hexose by sucrose synthase that is important for the early process of starch biosynthesis [38]. The developing kernel is an oxygen-limited tissue and the ATP level gradually decreased during the kernel filling [39]. PPDK is induced in response to hypoxia or anoxic stress and can also act glycolytically, which releases part of the energy to form ATP [23,40]. The PPDK reaction may have important roles in a storage metabolism by converting AMP to ATP in the hypoxic developing kernels. Several amyloplast enzymes exist in a large protein complex, which includes PPDK, AGPase, SSIIa, SSIIIa, BEIIa, and BEIIb, and this complex is responsible for coordinate regulation of starch synthesis [41]. Indeed, the expression of the complex members AGPS1, AGPS2, AGPL1, AGPL3, AGPL4, SSIIa, SSIIIa, BEIIa, and BEIIb in *M14* endosperm were significantly reduced (Figure 7). Therefore, the mutation of PPDK might also affect the function of the large protein complex.

## 4. Materials and Methods

### 4.1. Plant Materials and Growth Conditions

The grains of *japonica* rice variety Kitaake were irradiated with ^60^Co-γ ray at the dose of 300 Gy. Mature grains of M1 plant were harvested respectively and dehusked with a rice huller to screen endosperm mutants. A floury endosperm mutant *M14* was identified. The *M14* and an *indica* variety Dular were hybridized to produce F_2_ population for map-based cloning. The WT and *M14* were planted at the experimental farm of Yangzhou University in May and harvested in September.

### 4.2. Microscopy

The samples were prepared following the method as described previously [13]. Briefly, mature seeds were transversely broken by two tweezers, coated with gold, and examined with a scanning electron microscope (SEM, S-4800, Hitachi, Tokyo, Japan). For the observation of compound starch granules, transverse sections of WT and *M14* kernels at 9 DAF were fixed overnight in 2% (*v*/*v*) glutaraldehyde. The samples were dehydrated in an alcohol series, embedded in LR White resin, and sectioned using an ultrathin microtome (EM UC7, Leica, Wetzla, Germany). Semi-thin sections were dried at 40 °C, stained with I_2_-KI, and observed with a light microscope (BX53, Olympus, Tokyo, Japan).

### 4.3. Measurement of Starch Properties

Mature rice grains were air-dried and dehulled. Brown rice was further ground with a mortar and passed through a 0.15 mm sieve. The total starch content in the flour was determined with a total starch assay kit (Megazyme, Wicklow, Ireland). The amylose content was measured by using an iodine-potassium iodide colorimetric method as described previously [42]. The chain length distribution of amylopectin was analyzed using a fluorophore-assisted capillary electrophoresis (FACE) method according to the previous report [43]. The thermal properties and pasting properties of starch were measured by differential scanning calorimetry (DSC, 200-F3, Netzsch, Selb, Germany) and rapid visco analyzer (RVA, 3D, Newport Scientific, Narrabeen, Australia), respectively, following the method as described previously [44].

### 4.4. Map-Based Cloning of the OsPPDKB Gene

The samples with floury endosperm phenotype were selected from the F_2_ population of *M14* (*japonica*) and Dular (*indica*) for map-based cloning. One-hundred-and-seventy genome-wide InDel markers showing polymorphism were selected (Supplemental Table S2). The sequences of markers for fine mapping are shown in Supplemental Table S3.

### 4.5. Vector Construction and Complementation Test

The WT *cyOsPPDKB* cDNA sequence was cloned by PCR and inserted into the binary vector pCUbi1390 to generate the transformation cassette pUbi:cyOsPPDKB for complementation. The plasmid pUbi:cyOsPPDKB was introduced into *calli* generated from *M14* by *Agrobacterium* strain EHA105. Sixteen independent positive transgenic lines were obtained.

### 4.6. RNA Extraction and qRT-PCR

Total RNA from various tissues of WT and *M14* was extracted using the OminiPlant RNA Kit (CWBIO, Beijing, China). The first-strand cDNA was reverse transcribed by priming with oligo (dT18) based on HiScript 1st Strand cDNA Synthesis Kit (Vazyme, Nanjing, China). The qRT-PCR was performed on a CFX Connect real-time PCR system (Bio-Rad, Hercules, CA, USA) using AceQ qPCR SYBR Green Master Mix Kit (Vazyme, Nanjing, China). The PCR procedure was carried out using the following program: 95 °C for 10 min, then 40 cycles of 95 °C for 15 s, 60 °C for 1 min. The rice *Actin1* was employed as an endogenous control. Quantitative gene expression was analyzed from three biological replicates by the 2^−ΔΔ*C*t^ method, where ΔΔ*C*_t_ = (C_t,__T__arget gene_ − C_t,__A__ctin1_) *_M14_* − (C_t,__T__arget gene_ − C_t,__A__ctin1_) _WT_ [45]. The primers of *OsPPDKB* and *Actin1* were listed in Supplemental Table S3. The primers detecting starch biosynthesis have been reported elsewhere [12].

### 4.7. Subcellular Localization Analysis of OsPPDKB

The two transcripts of *OsPPDKB* (*chOsPPDKB* and *cyOsPPDKB*) were cloned in the pCAMBIA1305-GFP vector to create 35s::chOsPPDKB-GFP and 35s::cyOsPPDKB-GFP. The two binary vectors were transformed into *Nicotiana benthamiana* leaves following the method as described previously [11]. Laser scanning confocal microscope (LSM710, Zeiss, Oberkochen, Germany) was used to detect the GFP fluorescent signals.

### 4.8. Protein Extraction and Immunoblot Analysis

In order to separate the endosperm tissue, the developing kernels of WT and *M14* at 9 DAF were harvested, and the embryo and pericarp were removed subsequently. Proteins were extracted following the method as described previously [43], and transferred electrophoretically to nitrocellulose (NC) membrane after SDS-PAGE. The NC membrane was incubated in blocking buffer adding antibodies and was detected with ECL Plus reagent. Antibodies against GBSSI, SSI, BEI, BEIIa and BEIIb were described previously [46].

## 5. Conclusions

A floury endosperm mutant *M14* was isolated from Kitaake grains irradiated with 300 Gy ^60^Co-γ ray. Map-based cloning analysis showed that the floury endosperm phenotype was controlled by *cyOsPPDKB.* Abundant single and abnormal starch granules were observed in *M14* endosperm. Compared with WT, amylopectin short branch-chains with DP 6–12 increased, but intermediate branch-chains with DP 13–50 decreased in *M14*. The *M14* starch had a significantly lower gelatinization temperature and peak, hot and final viscosities. The chloroplast-localized PPDK highly expressed in leaf and leaf sheath and the cytoplasm-localized PPDK exhibited a high expression in developing endosperm. The expression of starch synthesis-related genes was significantly altered in *M14*. The substitution of single amino acid in *M14* affects the catalysis of PPDK on the reaction of Pyruvate to PEP, leading to the change of starch metabolism and structure through regulating the expression of starch synthesis-related enzymes in rice endosperm. This study contributed to an understanding of the role of PPDK in starch metabolism and structure and provided some information for quality breeding of rice.

## Figures and Tables

**Table 1:** Thermal properties of starches ^a^.

	*To* (°C) ^b^	*Tp* (°C) ^b^	*Tc* (°C) ^b^	Δ*T* (°C) ^b^	ΔH (J/g) ^b^
WT	60.4 ± 0.2	67.9 ± 0.1	74.0 ± 0.1	9.2 ± 0.2	13.7 ± 0.2
*M14*	57.7 ± 0.2 **	65.7 ± 0.3 **	72.1 ± 0.1 **	8.8 ± 0.3	14.4 ± 0.3 *

^a^ Data are given as means ± SD (*n* = 2). ** and * indicate significant differences between WT and *M14* at *p* < 0.01 and *p* < 0.05 by Student’s *t* test. ^b^ *To*, gelatinization onset temperature; *Tp*, gelatinization peak temperature; *Tc*, gelatinization conclusion temperature; Δ*T*, gelatinization temperature range (*T_c_*–*T_o_*); Δ*H*, gelatinization enthalpy.

## Supplementary Materials

The following are available online at http://www.mdpi.com/1422-0067/19/8/2268/s1.
