# Supplementary material for: A Novel Mutation of OsPPDKB, Encoding Pyruvate Orthophosphate Dikinase, Affects Metabolism and Structure of Starch in the Rice Endosperm
Source: Int J Mol Sci. 2018 Aug 2;19(8):2268. doi: 10.3390/ijms19082268 (PMC6121672; doi:10.3390/ijms19082268)
Supplement: Supplementary file 1 [file ijms-19-02268-s001.zip › Supplemental Data/Supplemental Figures.docx]

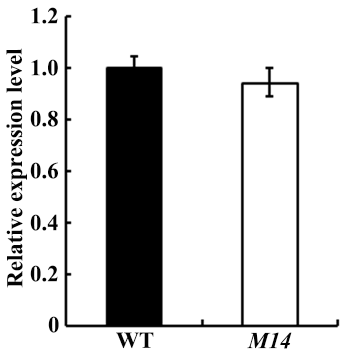


**Supplemental Figure S1.** Expression analysis of *cyOsPPDKB* in endosperm between WT and *M14* at 9 DAF.


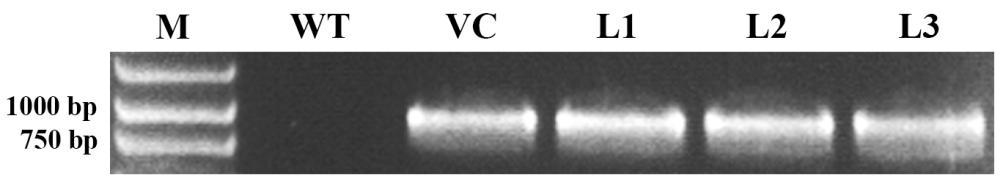

**Supplemental Figure S2**. The identification of positive transgenic lines by PCR amplification. Primer pairs in the vector are used to test the positive transgenic lines. M, marker; VC, vector control.


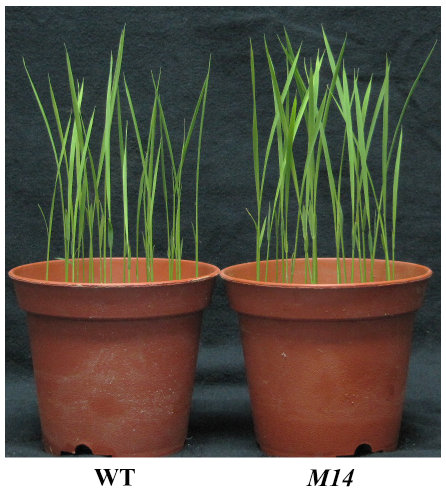


**Supplemental Figure S3.** Phenotypes of WT and *M14* seedlings at 10 days after germination.


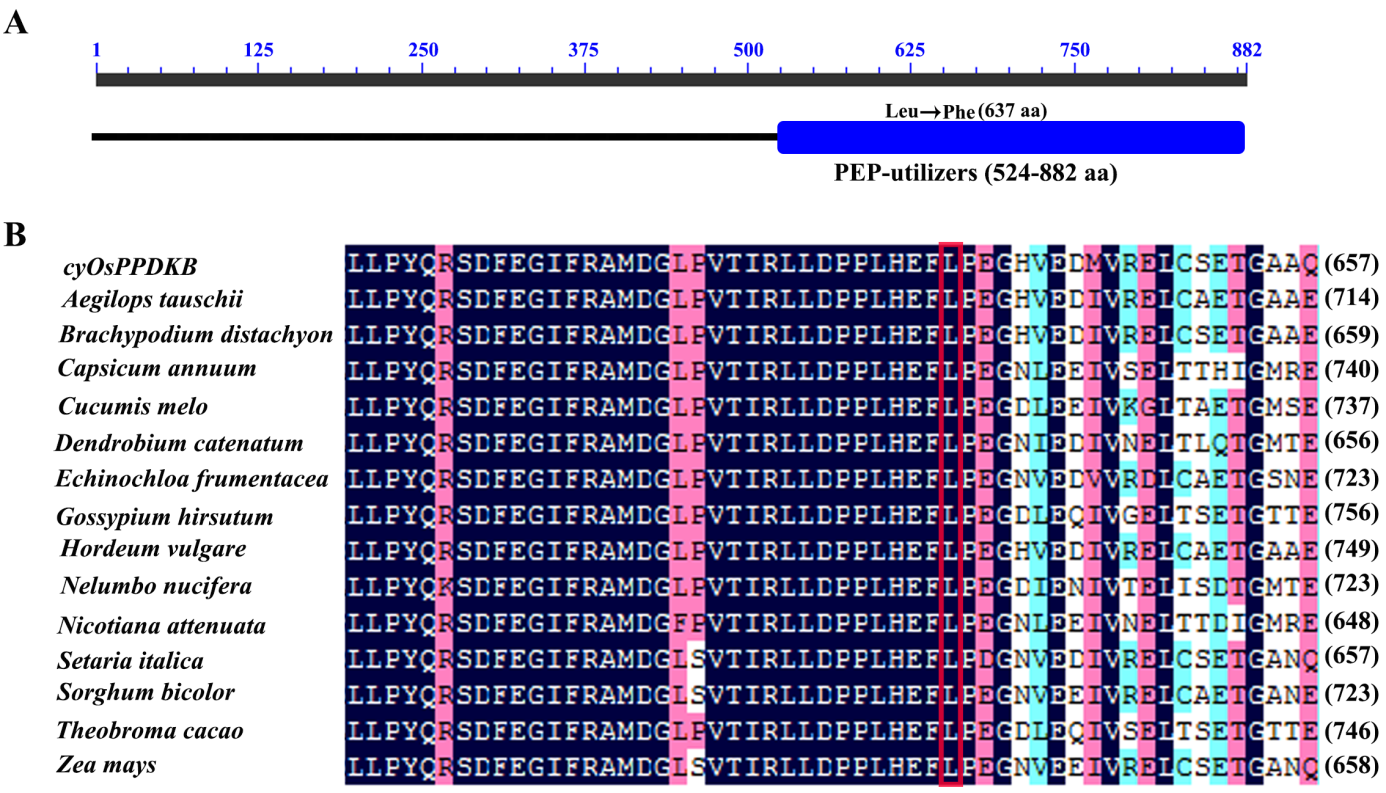


**Supplemental Figure S4.** Structure and sequence analyses of OsPPDKB protein. (A) Schematic domain structure of OsPPDKB. (B) Multiple amino acid sequence alignments of the mutated region of OsPPDKB protein. Highly conserved residues are highlighted with the same background. The mutation site is marked with a red box.
