# Supplementary material for: A Novel Mutation of OsPPDKB, Encoding Pyruvate Orthophosphate Dikinase, Affects Metabolism and Structure of Starch in the Rice Endosperm
Source: Int J Mol Sci. 2018 Aug 2;19(8):2268. doi: 10.3390/ijms19082268 (PMC6121672; doi:10.3390/ijms19082268)
Supplement: Supplementary file 1 [file ijms-19-02268-s001.zip › Supplemental Data/Supplementary Table S1.docx]

**Table S1**  Pasting properties of starches.^a^

|  | PV (mPa s) ^b^ | HV (mPa s) ^b^ | FV (mPa s) ^b^ | SV (mPa s) ^b^ | PT (°C) ^b^ |
| --- | --- | --- | --- | --- | --- |
| WT | 2407±13 | 2411±11 | 2774±2 | 366±15 | 77.7±0.6 |
| *M14* | 2070±4^**^ | 1906±12^**^ | 2449±9^**^ | 379±13 | 78.5±0.6 |

^a^ Data are given as means ± SD (n = 2). ** indicate significant differences between WT and *M14* at *P* < 0.01 by Student’s *t* test.

^b^ PV, peak viscosity; HV, hot viscosity; FV, final viscosity; SV, setback viscosity (FV-HV); PT, peak temperature.
