# Supplementary material for: A Novel Mutation of OsPPDKB, Encoding Pyruvate Orthophosphate Dikinase, Affects Metabolism and Structure of Starch in the Rice Endosperm
Source: Int J Mol Sci. 2018 Aug 2;19(8):2268. doi: 10.3390/ijms19082268 (PMC6121672; doi:10.3390/ijms19082268)
Supplement: Supplementary file 1 [file ijms-19-02268-s001.zip › Supplemental Data/Supplementary Table S3.docx]

**Table S3** Primers used in this study.

| **Usage** | **Primer Name** | **Sequence (**5' to 3') |
| --- | --- | --- |
| **Fine mapping** | IZ5-1-F | GCAGGAGATGAAAACGAGCA |
|  | IZ5-1-R | ATCCGTATCCTATCCGCACC |
|  | IZ5-2-F | GCACAAACGAGTCAGTAGCC |
|  | IZ5-2-R | GGAGCCATGATGTCCACTCT |
|  | IZ5-3-F | GTTAGAGATAAGAGTCGTGTCCG |
|  | IZ5-3-R | TCGGACGTCTCTCTAACAGC |
|  | IZ5-4-F | CCTCCGGGCTGATAGTACTT |
|  | IZ5-4-R | CCGGGCTGATAATACTTGTCG |
|  | IZ5-5-F | CTTCTAGCCCATTTGTGCGA |
|  | IZ5-5-R | GGGGAGAAGTGACGGGATTT |
|  | IZ5-6-F | CCGAGTCAATCTAGCCACCT |
|  | IZ5-6-R | GAGTGGCAGGCTTAATGTGG |
|  | IZ5-7-F | GTTCTGAGATTTCGGTCTGCC |
|  | IZ5-7-R | AAGGGTCACAGCATCAAAGC |
|  | Z5-12-F | AATTGGTCTAAATGGCAGCA |
|  | Z5-12-R | AGGATAATCTGCATATTGGGATA |
| **Genotyping** | 1390-F | ACGAGTCTAACGGACACCAAC |
|  | 1390-R | AGCATCTCTGTATATGCATC |
| **Binary vector construction** | 1390-cyOsPPDK-PstI-F | TCTGCACTAGGTACCTGCAGATGGCTCCGGCTCAATGTG |
|  | 1390-cyOsPPDK-BamHI-F | GAATTCCCGGGGATCCGAGGAGCACCTGAGCTGC |
|  | 35s::chOsPPDKB:GFP-BamHI-F | AGGACCGGTCCCGGGGGATCCATGCCGTCGGTTTCGAGG |
|  | 35s::cyOsPPDKB:GFP-BamHI-F | AGGACCGGTCCCGGGGGATCCATGGCTCCGGCTCAATGTG |
|  | 35s::OsPPDKB:GFP-BamHI-R | GCCCTTGCTCACCATGGATCCGAGGAGCACCTGAGCTGC |
| **quantitative RT-PCR** | P1-F | CGTGATCGCACCTCGCC |
|  | P1-R | TCTCCCCTCCAACTCCAAGC |
|  | P2-F | GAGCACCACACTTCACCAG |
|  | P2-R | GCCGAAGTGGAACACCCTC |
|  | qRT-Actin1-F | CCAAGGCCAATCGTGAGAAGA |
|  | qRT-Actin1-R | AATCAGTGAGATCACGCCCAG |
